# Secondary Malignancy in Giant Cell Tumor: A Single-Center Study

**DOI:** 10.3390/curroncol29060324

**Published:** 2022-06-02

**Authors:** Min Wook Joo, Yong-Suk Lee, Hong Sik Park, Yang-Guk Chung, Chiyoung Yoon

**Affiliations:** 1 Department of Orthopaedic Surgery, St. Vincent’s Hospital, College of Medicine, The Catholic University of Korea, 222, Banpo-daero, Seocho-gu, Seoul 06591, Korea; mwjoo@catholic.ac.kr (M.W.J.); cyyoon13@gmail.com (C.Y.); 2 Department of Orthopaedic Surgery, Incheon St. Mary’s Hospital, College of Medicine, The Catholic University of Korea, 222, Banpo-daero, Seocho-gu, Seoul 06591, Korea; 3 Department of Hospital Pathology, St. Vincent’s Hospital, College of Medicine, The Catholic University of Korea, 222, Banpo-daero, Seocho-gu, Seoul 06591, Korea; jjic2s@naver.com; 4 Department of Orthopaedic Surgery, Seoul St. Mary’s Hospital, College of Medicine, The Catholic University of Korea, 222, Banpo-daero, Seocho-gu, Seoul 06591, Korea; ygchung@catholic.ac.kr

**Keywords:** giant cell tumor of bone, malignancy in giant cell tumor, secondary malignancy, malignant transformation, radiotherapy, recurrence, prognosis

## Abstract

Giant cell tumor of bone (GCTB) undergoes a sarcomatous transformation. Secondary malignancy in giant cell tumor (MGCT) is associated with radiotherapy and has a dismal prognosis. We reviewed medical records to investigate the clinicopathological characteristics and prognosis of MGCT patients. The enrollment criterion was high-grade spindle-cell sarcoma, which developed at the site of prior GCTB treatment. Twelve patients were analyzed: six females and six males. The median age was 42.5 years. Benign recurrence occurred in five GCTB patients not treated with radiotherapy. No pulmonary implants were observed. The median latency to the malignant transformation was 63 months. Nine patients were AJCC stage IIB, and three were stage IVA. The median follow-up period after malignant transformation was 62.5 months. Five patients developed local recurrence, and six had distant metastasis. Five-year overall recurrence and metastasis-free survival rates were 61.9%, 66.7%, and 58.3%, respectively. Initial metastasis was a predictive factor for overall survival. Benign local recurrence of GCTB was also a negative factor for metastasis-free survival of MGCT patients. Differences in overall survival according to benign recurrence also showed a tendency toward significance. In our series, secondary MGCT did not occur after radiotherapy. The prognosis was better than previous findings. Benign recurrence of GCTB could reflect the prognosis of MGCT.

## 1. Introduction

Giant cell tumor of bone (GCTB) is a benign locally aggressive neoplasm with an incidence that varies according to the geographic region. Although it is a benign lesion, pulmonary metastasis can develop, which is known as pulmonary implants [1]. GCTB may uncommonly undergo sarcomatous transformation [2].

The term “malignant giant cell tumor” first appeared in the 1930s [3] and was used to describe sarcoma arising from a giant cell tumor [4]. However, the term frequently led to confusion, resulting in the inclusion of many giant cell-rich sarcomas in this category, which were not associated with GCTB [4,5]. In 2001, to resolve the confusion, the term “malignancy in giant cell tumor (MGCT)” was adopted [6]. MGCT is categorized into two subtypes based on the World Health Organization (WHO) classification [7]. Recent studies define primary MGCT as a type of tumor where high-grade sarcoma components appear beside benign GCTB components at the initial diagnosis, whereas secondary MGCT is defined as high-grade sarcoma components that develop in previously treated GCTB [4,5,8,9]. Therefore, a previous history of GCTB and the information from local recurrence were the main differentiating points between secondary MGCT and different malignancies [5,10].

Previous studies have reported that most MGCT was secondary and developed normally after radiotherapy, but it can follow surgery without adjuvant radiation therapy [6,10,11]. It was suggested that irradiation affected malignant transformation and decreased the latency interval [4]. Most studies report a dismal prognosis for patients with MGCT [4,5,9]. However, there currently is no consensus on the appropriate treatment [8].

Unfortunately, most published studies involving a relatively large number of cases failed to collect patient data based on a consistent definition and subclassification of MGCT [12], so it is not easy to analyze or compare the previous studies with each other. Therefore, we investigated the clinicopathological characteristics and prognostic factors of patients with secondary MGCT based on the recent diagnostic criteria.

## 2. Materials and Methods

This study was a retrospective medical record review of patients with histologically confirmed secondary MGCT who were surgically treated at our tertiary center from 1995 to 2018. The enrollment criterion was histologically proven high-grade spindle-cell sarcoma, which developed at the previous treatment site in patients with benign GCTB, reflecting the definitions in the two latest editions of the WHO classification of tumors [4,7,9,10,13,14,15,16,17].

This study (VC20RISI0093) was approved by the Catholic University of Korea St. Vincent’s Hospital Institutional Review Board, which dispensed with the need for informed consent, as this study was a retrospective and a minimal risk one, and any identifiable personal information was not collected. All methods and research were performed in accordance with the relevant guidelines and regulations of the ethics commission.

Clinical and radiological information was obtained. Data regarding gender, age, location, the Campanacci grade [18] of primary GCTB, initial treatment of the primary lesion, local recurrence or pulmonary implants of GCTB, latency to malignant transformation, lesion size of the MGCT at the initial presentation, the American Joint Cancer Committee (AJCC) [19,20] and Enneking stage [20] of MGCT, MGCT treatment, local recurrence or distant metastasis of the malignancy, the follow-up period, and oncologic outcomes were reviewed. The Campanacci grade [18] was determined as follows: Grade I lesions have a well-marginated border and intact cortex; Grade II tumors have relatively well-defined margins but no radiopaque rim, and the cortex is thinned and moderately expanded; Grade III describes a lesion with indistinct borders and cortical destruction. Lesion volume was calculated as π/6 × (length axis) × (width axis) × (height axis). In addition, the number of GCTB patients who were treated in our hospital during the same study period was investigated.

In patients with a latent interval of fewer than three years, existing histopathologic slides and new ones made using paraffin blocks were reviewed again by two experienced pathologists because secondary malignancies usually occur at least three years after the initial GCTB [21]. The latent period was defined as the period from the date of the first surgery for GCTB to the diagnosis of malignant transformation. In MGCT, local recurrence-free survival, distant metastasis-free survival, and overall survival were evaluated based on the intervals from the time of initial surgery for the malignant lesion to the time of the first local recurrence, the first distant metastasis, and death or final follow-up, respectively. The follow-up period was defined as the interval from the date of the malignant transformation diagnosis to the last follow-up.

The numerical limits for the categorization of each factor were chosen so that the *p*-value came out the least. The 5-year survival rates for MGCT were analyzed using the Kaplan–Meier method, and the log-rank test was used to compare the survival curves for univariate analysis. The impact of potential prognostic factors was assessed using the log-rank test by univariate analysis. A *p*-value of < 0.05 was considered significant. All statistical analyses were performed using SPSS 21.0 for Windows (SPSS Corporation, Chicago, IL, USA).

## 3. Results

A total of 12 cases were surgically treated and histologically confirmed as secondary MGCT at our tertiary center from 1995 to 2018. The patients were reviewed, and the details are presented in Table 1 and Table 2. During the study period, 143 patients with GCTB were managed in our institution. The study included six males and six females, with a median age of 42.5 years (range, 36–66 years) at the initial diagnosis of MGCT. Six lesions were located in the distal femur, three in the proximal tibia, two in the proximal femur, and one in the distal radius. Six patients with benign GCTB were assigned Campanacci grade [18] II radiologically and six were grade III. Surgical treatment for lesions entailed intralesional tumor removal in 11 patients. In addition, bone grafting and cementation were performed in six and three patients, respectively. The second patient listed in Table 1 had tumor resection and arthroplasty using an implant. Denosumab was not administered to any patient. Benign recurrent GCTB occurred in five cases. Among them, the seventh case in Table 1 relapsed three times. No patient had radiotherapy for benign GCTB and recurrent lesions. No pulmonary implants were observed.

The patients were diagnosed with MGCT and underwent surgical treatment in our tertiary center from 1995 to 2018. All patients presented with pain and the sixth patient in Table 1 visited our center following a pathologic fracture. All of the lesions developed in the extremities. The median latent period was 63 months (range, 7–240 months). The median main diameter of the MGCT lesions at presentation was 8.35 cm (range, 4.5–11.1 cm) and the volume was 199.1 cm^3^ (range, 50–968.11 cm^3^). Nine patients were AJCC stage [19,20] IIB and Enneking stage [20] IIA, and three were AJCC [19,20] stage IVA and Enneking stage [20] III. Nine patients demonstrated osteolytic lesions, and three showed osteoblastic lesions in plain radiographs. Magnetic resonance imaging revealed extraosseous extension in all patients. The seventh case in Table 1 carried a secondary aneurysmal bone cyst. Eleven patients underwent wide resection and limb reconstruction with endoprostheses, while the eighth case, presented in Table 1, had an amputation. Negative surgical margins were achieved in all patients. Ten patients underwent postoperative chemotherapy, among whom two also had preoperative chemotherapy. The first-line chemotherapeutic regimens included doxorubicin, ifosfamide, methotrexate, and cisplatin. The second-line chemotherapy was gemcitabine and docetaxel. Pulmonary metastasectomy was considered depending on resectability. Active surgical treatment was performed when local recurrence occurred in patients with no evidence of disease. No patients underwent radiation treatment for MGCT. The prognoses after the diagnosis of MGCT are summarized in Table 2. Local recurrences occurred in five patients, and the median local recurrence-free survival interval was 7 months (range, 5–202 months). Six patients had distant metastasis, and the median distant metastasis-free survival interval was 6.5 months (range, 0–38 months). The median follow-up period was 62.5 months (range, 6–294 months). Regarding the oncologic outcomes at the last follow-up, five patients were continuously disease-free, one had no evidence of disease, five died of the disease, and one died of other diseases.

**Figure 1:**
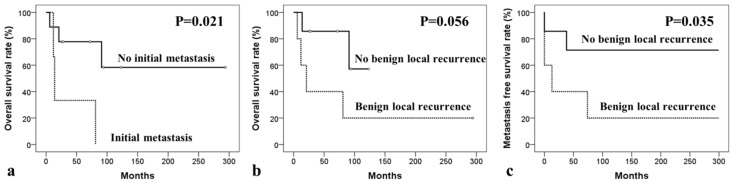
Kaplan–Meier survival curves of univariate analyses for secondary malignancy in giant cell tumor. Kaplan–Meier curves for overall survival according to (**a**) initial metastasis and (**b**) benign recurrence of giant cell tumor of bone, and for (**c**) metastasis-free survival depending on benign local recurrence.

In MGCT patients, 5-year overall survival rate (OSR), local recurrence-free survival rate (RFSR), and distant metastasis-free survival rate (MFSR) were 61.9%, 66.7%, and 58.3%, respectively. The analysis of potential prognostic factors for OSR, RFSR, and MFSR are presented in Table 3, Table 4 and Table 5, respectively. The difference in OSR according to initial metastasis (yes versus no) showed statistical significance (*p* = 0.021) and, depending upon the benign local recurrence of GCTB (yes versus no), showed a trend toward significance (*p* = 0.056). None of the potential factors had any impact on the RFSR. The difference in MFSR depending upon benign local recurrence (yes versus no) was significant (*p* = 0.035) (Figure 1).

## 4. Discussion

While it is generally known that GCTB constitutes 5% to 7% of all primary bone tumors and 20% of benign skeletal tumors [22,23], the incidence differs by regional groups. Overall, it seemed higher in Asian countries than in the Western population. Among all primary bone tumors, the incidence of GCTB was estimated at around 14% to 20% in China [24,25]. Interestingly, a Japanese cohort showed a low incidence of about 2% to 7% [26,27]. Although Sweden is a European country, the GCTB incidence was reported to be about 11% [28]. The incidence was reportedly greater than 30% in southern India [29], whereas it was around 6% in western India [30].

Although radiotherapy is known to induce malignant tumors [31,32] and most secondary MGCT cases have developed after radiation treatment in previous studies [10], none of the cases in this study were associated with radiation treatment. Currently, limited radiotherapy data are available for benign GCTB [11] because the treatment is indicated for locations where curative surgery is unfeasible, such as the spine or sacrum, and for aggressive and recurrent tumors [32]. However, several studies [31,32,33,34,35,36] have demonstrated clinical results. According to the Western literature [32,33,34], radiotherapy was often performed for GCTB [37] and resulted in favorable local control rates, whereas the risk of post-radiation malignancies was a concern [31,32,33,34]. In a retrospective review, 26 lesions, 9% of the total cases, with a high risk of local recurrence treated at an institution from 1972 to 1996 reported a 77% local control rate (LCR) and the development of one post-radiation sarcoma 22 years after radiotherapy [32]. Another study investigating 122 consecutive patients with unresectable GCTB between 1985 and 2007 showed an 85% LCR and the occurrence of two malignant transformations during a median follow-up of 58 months [33]. In another study involving 34 patients from 1973 to 2008, an LCR of 81% was reported 15 years after radiation treatment, and one secondary malignancy developed 52 months after radiotherapy [34].

Previous studies with a large case series revealed that most secondary MGCTs were not associated with radiotherapy in an Asian population [38] even though most of the cases were post-radiation sarcoma in a Western population [10]. Radiotherapy appears to be rarely used for benign GCTB in Asian countries. We searched the MEDLINE, Embase, and Cochrane databases in May 2020 using the terms “giant cell tumor” AND (“radiation” OR “radiotherapy”) and found only two relevant studies published in Asian countries. Radiation treatment was used for five of 35 patients with extremity disease in one study [35] and for 18 of 22 cases with GCTB in the axial skeleton in another [36]. In contrast, two relatively large case series from China reported that radiation treatment was not applied in 621 patients with GCTB in extremities [24] and 208 other cases [25].

While cases of malignant transformation of GCTB during denosumab treatment have been reported, they are rare, and the causality of the relationship between denosumab and sarcomatous change cannot be determined [39]. The potential mechanisms of malignant change of GCTB after denosumab treatment are probably related to its actions against the receptor activator of nuclear factor-kappa B ligand (RANKL) [40]. Three hypotheses are proposed even though the definite molecular background is not determined [39]. As RANKL is critical in the development of lymphocytes and the organogenesis of lymph nodes, denosumab may influence immunity and inflammation [41,42,43]. The risk of new malignancy could increase as a result of immunosuppression by RANKL inhibition. Secondly, RANKL expression increases the level of nuclear factor IB [44], a transcription factor exhibiting tumor suppression effects through downregulating susceptibility to nuclear oncogenes in osteosarcoma [45]. Therefore, RANKL inhibition could induce osteosarcoma development. Lastly, RANKL upregulates the Semaphorin 3A gene level in osteosarcoma [44], and its deletion could induce the bizarre growth of cartilage and bone [46,47]. Consequently, RANKL inhibition by denosumab possibly leads to the abnormal differentiation of osteoblasts and osteosarcoma tumorigenesis through Semaphorin 3A. Meanwhile, the recent phase II study with 526 patients who received at least one dose of denosumab reported that it was clear that sarcomatous transformation developed since using the regimen only in four cases and the incidence of secondary malignancy was lower than historical rates [48]. In this study, no patients were administered denosumab.

Pulmonary implants were observed in 2% of the patients with GCTB at a mean duration of three to four years after the initial diagnosis [49]. In general, such lung metastasis developed in benign GCTB of unusual anatomical sites such as the spine or the pelvis and rarely occurred at the initial presentation [50]. As local recurrence of GCTB is a known risk factor for pulmonary implants [1], the biologic activity of GCTB may be related to lung metastasis. However, no pulmonary implants were observed, although some local recurrences were diagnosed in the current study. Therefore, whether a pulmonary implant is a risk factor for malignant transformation is unclear.

Although the number of GCTB and secondary MGCT patients treated at our institution during the study period were 143 and 12, respectively, the incidence of malignant transformation of GCTB cannot be estimated at 8.4% because 11 of all 12 MGCT patients were referred from other primary or general hospitals. As only one patient was diagnosed with secondary MGCT after GCTB treatment at our institution, we believe that an incidence of <0.7% could be more reliable. Previous studies have reported that the incidence of non-post-radiation secondary MGCT was below 0.7% [4,12,13,22,38].

**Figure 2:**
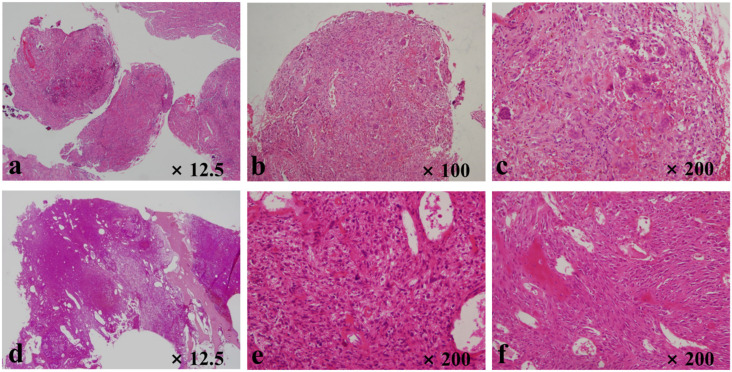
Histopathologic evaluation of the sixth case listed in Table 1. The slides of benign giant cell tumor of bone show (**a**) fibrous tissues with giant cells, (**b**) giant cells and histiocytes, and (**c**) spindle-cell lesions with giant cells and histiocytes. Slides related to secondary malignancy in giant cell tumors demonstrate (**d**) lesion filling most of the intramedullary space in the distal femur, (**e**) malignant spindle cells with a few giant cells, and (**f**) malignant osteoids.

In our study, MGCT developed at a median interval of seven years and four months after the first treatment for benign GCTB. Several previous studies have reported latent periods of 1.8 to 36 years for the malignant transformation of a benign lesion after surgery alone [4,14,15,38] and 4 to 42 years after radiation treatment [4,14,15,51], which suggests no significant difference in the latency interval depending upon radiotherapy. Nevertheless, a recent Western study [4] demonstrated that the mean latent period was 9 years in patients who underwent radiation therapy and 18 years in those who did not and proposed that irradiation would have an impact on sarcomatous change and shorten its latent period. However, an Asian study reported a short mean latent interval of 42 months in 3 patients out of 110 with GCTB. Among them, one developed MGCT nine months after surgery without previous exposure to irradiation, and one case developed seven months postoperatively in the current study (Figure 2) [38]. To exclude the possibility of malignancy involving the original lesion, pathologic slides were repetitively reviewed by two experienced pathologists in the current study. As the lesions were diagnosed with entire specimens obtained by extended curettage, the histologic confirmations were unlikely to be inaccurate. Several recent studies have demonstrated that the H3F3A mutation might contribute to distinguishing GCTB-related tumors from those that are giant-cell-rich [10,52,53,54]. However, there are differences in the frequency of H3F3A mutations found in previous reports; these mutations were identified in 69–100% of GCTB [53,54,55,56,57,58,59,60,61,62]. One study [54] suggested that diagnosis of GCTB without the H3F3A alteration should be confirmed with considerable caution. However, another study [62] described that all diagnoses were made in conjoint assessment by radiologists, pathologists, and orthopedic surgeons in a multidisciplinary team meeting despite the advancement of ancillary diagnostic tests and that the original diagnoses were not changed in the cases where no mutation was confirmed. Even in primary and secondary MGCT, H3F3A mutations can or cannot be found [63,64]. Therefore, the analysis of H3F3A cannot confirm the diagnosis of GCTB and cannot differentiate primary and secondary MGCT completely. As we have thoroughly reviewed our cases, it was considered that the result of the mutation test could rather undermine the reliability of the diagnosis based on a multidisciplinary team approach.

Although the resection margins were regarded as negative in all 12 MGCT patients in the current study, 5 patients developed local relapses. Four of them underwent limb-salvage operations. A previous study [18] demonstrated no local recurrence after surgical treatment in two non-post-radiation secondary MGCT cases. Another study reported one local relapse among six patients [4]. The local recurrence rate seems relatively high in our study. However, it would be difficult to directly compare the rates from different studies because the number of non-post-radiation secondary MGCT cases in the studies was small, the surgical methods might differ, and the individual patients showed different survival periods [4,18].

Due to the rarity of the disease, and the unclear definition and subclassification of MGCT, the prognosis has yet to be established. Most studies reported a dismal prognosis regardless of primary or secondary MGCT [5,9]. The prognosis for secondary MGCT was unfavorable, compared with primary MGCT in previous studies from Western countries [4,14,65]. Exceptionally, a study [9] reported a 5-year survival rate of 50% in both the primary and secondary MGCT groups. Another study demonstrated that the 5-year survival rate of patients with primary malignancy was 87% and implied that MGCT behaved like a low- to intermediate-grade sarcoma [12], which was contrary to other studies. However, the distinction between primary and secondary MGCT was practically vague [12], although the study was regarded as relatively well-designed, compared with previous ones [10].

The poor prognosis of secondary MGCT following radiotherapy is believed to be attributed to the unfavorable tumor location where radiation treatment is a unique option [4]. Lymphatic destruction, vascular deficiency, or fibrosis after radiotherapy could also cover malignant cells from the immune system [66], which may result in more aggressive and poorly differentiated secondary lesions [67]. As no patient in the current study received radiotherapy, their oncologic outcome may have been relatively favorable. Nonetheless, a 5-year survival rate of 61.9% is unlikely to be explained by the influence of radiation alone. Given the differences in the incidence of GCTB according to regions, latency depending upon ethnicity, and prognosis reported in previous studies, ethnic factors may play important roles in the development of MGCT and its prognosis.

An analysis of the Surveillance, Epidemiology, and End Results (SEER) database of patients with MGCT reported that the age at diagnosis, tumor size and extension, and radiation treatment were prognostic factors for overall survival [8]. Age at diagnosis and tumor size were not significant prognostic factors, and tumor extension was not evaluated in the current study. Local recurrence of benign GCTB was a significant prognostic factor for MFSR. In addition, the difference in the OSR depending upon the benign recurrence of GCTB was almost significant.

There is currently no comprehensive agreement on MGCT management [8]. Curative surgery is generally considered when it is feasible [4]. The efficacy of chemotherapy in MGCT is unclear [4]; however, several studies [14,65,68] have reported that the use of chemotherapy offered some benefits. A previous report [9] demonstrated that the difference in 5-year survival rates was statistically insignificant between the groups that were treated by surgery alone and the combination of surgery and chemotherapy. Another study showed that adjuvant chemotherapy as a salvage procedure following surgery with an inadequate margin did not result in any obvious advantage [14]. In this study, chemotherapy was also not a statistically significant prognostic factor. Another recent study reported that resected tumors in three patients out of four who were administered preoperative chemotherapy based on an osteosarcoma protocol showed excellent necrosis rates [9]. Radiation treatment was frequently used to treat MGCT in the past [14,69]. However, the preference has declined lately [8]. In contrast to findings from Western countries, radiotherapy does not appear to be used readily for managing post-radiation sarcomas in Asian populations [31].

There were several limitations to this study. Inevitably, this retrospective analysis could not exclude inclusion bias. Its statistical power, especially for prognostic factor analysis, was limited by the small number of cases as the incidence of secondary MGCT was extremely low, with approximately 1% to 5% of the patients with GCTB undergoing sarcomatous transformation to secondary MGCT in four large case series [10]. Nevertheless, the current study could be worthwhile, as these patients are different from those in existing studies because they did not receive radiation treatment at all, and the cases were only extremity lesions.

## 5. Conclusions

In our series, the occurrence of secondary MGCT did not follow radiotherapy, contrary to reports in Western literature. The prognosis was better when compared with findings reported in previous studies. Local recurrence of benign GCTB before malignant transformation could reflect the prognosis of MGCT. Further studies with a large number of cases are mandatory, especially to elucidate the ethnic differences in the development and prognosis of MGCT.

## Figures and Tables

**Table 1:** Patient characteristics.

Location	Primary Benign Giant Cell Tumor			Latent Period (Month)	Malignancy in Giant Cell Tumor					
	Campanacci Grade	Surgical Method	Local Recurrence		Patient No./Gender/Age (Years)	Size (cm)	Radiologic Feature	Stage (AJCC/ Enneking)	Surgery Type	Histology/ Subtype
PT	3	Cu and Ce	0	90	1/M/53	10.3 × 10.3 × 3	Osteolytic	IIB/IIA	LS	OSA/ conventional
PF	2	Re and AP	1	180	2/M/41	10.5 × 10 × 4	Osteolytic	IVA/III	LS	OSA
PT	3	Cu and Ce	0	123	3/M/41	10.8 × 10.5 × 8.3	Osteolytic	IIB/IIA	LS	UPS
DF	2	Cu and BG	0	150	4/M/41	8.5 × 6 × 4	Osteoblastic	IIB/IIA	LS	OSA/ osteoblastic
DF	2	Cu and Ce	1	25	5/F/40	5 × 5 × 2	Osteolytic	IIB/IIA	LS	OSA
DF	3	Cu	0	7	6/F/62	11.1 × 7.4 × 3.7	Osteolytic	IIB/IIA	LS	OSA
PT	3	Cu and BG	3	240	7/F/66	9.0 × 6.5 × 4.3	Osteolytic ABC change	IIB/IIA	LS	OSA
DF	2	Cu	0	36	8/F/50	5.8 × 6.5 × 3.3	Osteoblastic	IVA/III	Amp	OSA/ osteoblastic
DR	3	Cu and BG	1	24	9/F/40	4.5 × 4.0 × 3.0	Osteolytic	IIB/IIA	LS	OSA/ fibroblastic
DF	3	Cu	0	126	10/F/36	5.1 × 3 × 4	Osteolytic	IIB/IIA	LS	OSA/ fibroblastic
PF	2	Cu and BG	1	27	11/M/44	8.2 × 6.4 × 3.7	Osteoblastic	IVA/III	LS	OSA/ osteoblastic
DF	2	Cu and BG	0	33	12/M/59	6.0 × 5.5 × 4.3	Osteolytic	IIB/IIA	LS	OSA/mixed type

PT, proximal tibia; PF, proximal femur; DF, distal femur; DR, distal radius; Cu, curettage; Ce, cementation; Re, resection; AP, arthroplasty; BG, bone graft; LS, limb salvage; Amp, amputation; OSA, osteosarcoma; UPS, undifferentiated pleomorphic sarcoma.

**Table 2:** Prognosis after malignant transformation of giant cell tumor.

Patient No./ Gender/Age (Years)	Local Recurrence		Distant Metastasis			Follow-Up Period (Months)	Oncologic Outcome
		Interval from Diagnosis (Months)		Site	Interval from Diagnosis (months)		
1/M/53	Yes	38	Yes	Lung	38	91	DOD
2/M/41	Yes	7	Yes	Lung	0	81	DOD
3/M/41	No	-	No	-	-	28	CDF
4/M/41	No	-	No	-	-	72	CDF
5/F/40	Yes	202	Yes	Lung	74	294	NED
6/F/62	No	-	No	-	-	94	CDF
7/F/66	No	-	No	-	-	6	DOAD
8/F/50	Yes	5	Yes	Lung	0	14	DOD
9/F/40	Yes	5	Yes	Lung	13	21	DOD
10/F/36	No	-	No	-	-	53	CDF
11/M/44	No	-	Yes	Lung	0	12	DOD
12/M/59	No	-	No	-	-	26	CDF

DOD, died of disease; CDF, continuously disease-free; NED, no evidence of disease; DOAD, died of another disease.

**Table 3:** Statistical analysis of prognostic factors for overall survival.

Factors		Univariate Analysis		
		*n* (%)	5-Year OSR (%)	*p* Value
Gender	Male	6 (50%)	83.3	0.888
	Female	6 (50%)	50	
Age	≥50 years	5 (41.7%)	60	0.641
	<50 years	7 (58.3%)	71.4	
Campanacci grade	2	6 (50%)	67.7	0.837
	3	6 (50%)	67.7	
Benign local recurrence	No	7 (58.3%)	85.7	0.056
	Yes	5 (41.7%)	40	
Latent period	≥10 years	5 (41.7%)	80	0.746
	<10 years	7 (58.3%)	57.1	
Main diameter	≥8 cm	7 (58.3%)	71.4	0.541
	<8 cm	5 (41.7%)	60	
Volume	≥250 cm^3^	5 (41.7%)	80	0.801
	<250 cm^3^	7 (58.3%)	57.1	
MGCT local recurrence	No	7 (41.7%)	71.4	0.238
	Yes	5 (58.3%)	60	
Initial metastasis	No	9 (75%)	77.8	0.021 *
	Yes	3 (25%)	33.3	
Chemotherapy	No	2 (16.7%)	100	0.174
	Yes	10 (83.3%)	60	

MGCT, malignancy in giant cell tumor; OSR, overall survival rate; * statistically significant.

**Table 4:** Statistical analysis of prognostic factors for local recurrence-free survival.

Factors		Univariate Analysis		
		*n* (%)	5-Year RFSR (%)	*p* Value
Gender	Male	6 (50%)	100	0.574
	Female	6 (50%)	60	
Age	≥50 years	5 (41.7%)	75	0.934
	<50 years	7 (58.3%)	83.3	
Campanacci grade	2	6 (50%)	80	0.471
	3	6 (50%)	80	
Benign local recurrence	No	7 (58.3%)	85.7	0.09
	Yes	5 (41.7%)	66.7	
Latent period	≥10 years	5 (58.3%)	100	0.313
	<10 years	7 (41.7%)	66.7	
Main diameter	≥8 cm	7 (41.7%)	66.7	0.271
	<8 cm	5 (58.3%)	100	
Volume	≥250 cm^3^	5 (58.3%)	100	0.888
	<250 cm^3^	7 (41.7%)	66.7	
Chemotherapy	No	2 (16.7%)	100	0.125
	Yes	10 (83.3%)	75	

RFSR, local recurrence-free survival rate.

**Table 5:** Statistical analysis of prognostic factors for distant metastasis-free survival.

Factors		Univariate Analysis		
		*n* (%)	5-Year MFSR (%)	*p* Value
Gender	Male	6 (50%)	83.3	0.888
	Female	6 (50%)	60	
Age	≥50 years	5 (41.7%)	75	0.814
	<50 years	7 (58.3%)	71.4	
Campanacci grade	2	6 (50%)	67.7	0.302
	3	6 (50%)	80	
Benign local recurrence	No	7 (58.3%)	85.7	0.035 *
	Yes	5 (41.7%)	50	
Latent period	≥10 years	5 (58.3%)	100	0.22
	<10 years	7 (41.7%)	57.1	
Main diameter	≥8 cm	7 (58.3%)	57.1	0.663
	<8 cm	5 (41.7%)	60	
Volume	≥250 cm^3^	5 (58.3%)	60	0.590
	<250 cm^3^	7 (41.7%)	57.1	
Chemotherapy	No	2 (16.7%)	100	0.109
	Yes	10 (83.3%)	66.7	

MGCT, malignancy in giant cell tumor; MFSR, distant metastasis-free survival rate; * statistically significant.

## Data Availability

The data presented in this study are available on request from the corresponding author.
